# Energy, Exergy, Exergoeconomic and Emergy-Based Exergoeconomic (Emergoeconomic) Analyses of a Biomass Combustion Waste Heat Recovery Organic Rankine Cycle

**DOI:** 10.3390/e24020209

**Published:** 2022-01-28

**Authors:** Saeed Khojaste Effatpanah, Mohammad Hossein Ahmadi, Seyed Hamid Delbari, Giulio Lorenzini

**Affiliations:** 1 Faculty of Mechanical and Mechatronics Engineering, Shahrood University of Technology, Shahrood 3619995161, Iran; Saeed.khojaste@shahroodut.ac.ir (S.K.E.); mohammadhosein.ahmadi@gmail.com (M.H.A.); 2 Department of Renewable Energies and Environmental, Faculty of New Sciences and Technologies, University of Tehran, Tehran 1439957131, Iran; hamiddelbari@ut.ac.ir; 3 Department of Engineering and Architecture, University of Parma, Parco Area Delle Scienze, 181/A, 43124 Parma, Italy

**Keywords:** waste heat recovery (WHR), organic Rankine cycle (ORC), exergoeconomic, emergoeconomic, sustainability

## Abstract

In recent decades, there has been an increasing trend toward the technical development of efficient energy system assessment tools owing to the growing energy demand and subsequent greenhouse gas emissions. Accordingly, in this paper, a comprehensive emergy-based exergoeconomic (emergoeconomic) method has been developed to study the biomass combustion waste heat recovery organic Rankine cycle (BCWHR-ORC), taking into account thermodynamics, economics, and sustainability aspects. To this end, the system was formulated in Engineering Equation Solver (EES) software, and then the exergy, exergoeconomic, and emergoeconomic analyses were conducted accordingly. The exergy analysis results revealed that the evaporator unit with 55.05 kilowatts and the turbine with 89.57% had the highest exergy destruction rate and exergy efficiency, respectively. Based on the exergoeconomic analysis, the cost per exergy unit (c), and the cost rate $\left(\dot{C}\right)$ of the output power of the system were calculated to be 24.13 USD/GJ and 14.19 USD/h, respectively. Next, by applying the emergoeconomic approach, the monetary emergy content of the system components and the flows were calculated to evaluate the system’s sustainability. Accordingly, the turbine was found to have the highest monetary emergy rate of capital investment, equal to $5.43\times 10^{12}\;\mathrm{sej}/h$, and an output power monetary emergy of $4.77\times 10^{4}\;\mathrm{sej}/J$. Finally, a sensitivity analysis was performed to investigate the system’s overall performance characteristics from an exergoeconomic perspective, regarding the changes in the transformation coefficients (specific monetary emergy).

## 1. Introduction

Overexploitation of fossil fuels to meet the ever-increasing global energy demand triggers the exhaustion of conventional resources, global warming, air pollution, and ozone depletion [1]. At present, energy conservation and air pollution reduction measures have comprised the main strategies to address the above issues, given the high base population [2]. Waste heat recovery is a promising technology specifically tailored to this end, which has been practiced favorably in recent decades [3]. In this regard, the Organic Rankine Cycle (ORC), known to researchers since the 70s, is considered a potential reliable technology to recover heat efficiently from low to mid-temperature heat sources in sustainable energy utilization systems [4,5], as well as in conventional industrial processes [6,7].

The four main components of the basic ORC are the pump, evaporator, turbine, and condenser [8]. It offers several advantages, including lower operating temperatures and pressure ranges, lower O&M (Operation and Maintenance) costs, safe and autonomous operation, and simplicity, which cultivates interest in the technology [9,10]. Unlike the conventional Rankine cycle, ORC is widely used at micro (below 15 kW) and small (below 100 kW) scales [5,11], as well as in decentralized CHP (Combined Heat and Power) units [12,13]. Typically, the working fluid of an ORC is fluids with low boiling points (for instance, alcohols, ether, refrigerant, etc.), while conventional Rankine cycles operate with steam or water [14]. Utilizing a dry working fluid eliminates the need for superheating and reduces mechanical stresses, thereby lowering the O&M costs and extending the system’s lifetime [15].

ORC power plant coupling with waste heat from industrial processes, biomass incinerators, or geothermal energy has been successfully commercialized. According to the latest report provided in ref. [16], the total capacity of ORC power plants installed at 1754 sites exceeds 2.7 GW, with a biomass share of nearly 11% (301 MW) from 332 sites. It should be noted that most of the biomass combustion waste heat recovery ORC (BCWHR-ORC) units are installed at multi-purpose manufacturing facilities [17].

Numerous low to mid-temperature energy sources such as solar energy [18,19], geothermal energy [20,21], biomass [22,23], and industrial waste heat [24,25] generate clean electricity that reduces environmental pollution while improving energy efficiency. The selection of working fluid [26], operational conditions [27], and integration of ORC with different processes [28] significantly affect the net power output of the system. Additionally, employing optimization methods provides a satisfactory trade-off between the thermal efficiency, rate of the recovered waste heat, and economic factors [29].

A variety of research has been carried out on ORC performance improvement so that they can be widely utilized in waste heat recovery applications over various temperature ranges. In this light, Feng et al. [30] introduced an irreversible regenerative ORC, and optimized the output power and efficiency while keeping the surface area available for heat transfer constant in all heat exchangers. In another study, Braimakis et al. [31] optimized three improved designs of regenerative ORC using various working fluids from an energy analysis perspective. The coupling of a vapor compression cycle with an ORC for heat recovery purposes has been investigated by Zhar et al. [32]; through a multi-objective optimization of their parametric analysis, the proposed system’s return of investment was calculated to be 6.3 years. The implementation of ORC to recover the waste heat capacity in the aluminum industry has been studied by Dokl et al. [33]. The authors concluded that up to 830 kW of electrical power could be generated using the waste heat produced by the Slovenian aluminum manufacturer they investigated. In an experimental study, Wang et al. [34] achieved a turbine isentropic efficiency of 88.6% in a 300 kW low-temperature waste heat recovery ORC. This efficiency corresponded to a waste heat source temperature of 121 °C. Ming et al. [35] extracted electrical power from the waste heat produced from an aluminum melting process using an ORC. Having assessed the thermodynamic performance of the proposed design, they suggested utilizing such systems in aluminum electrolysis facilities as an effective energy conservation measure.

Recently, few researchers have investigated the applicability of mid to high-temperature biomass combustion waste heat recovery with ORC. For instance, Georgousopoulos et al. [36] carried out a thermodynamic and techno-economic assessment of an ORC coupled with an integrated gasification combined cycle under three different scenarios to optimize waste heat recovery. They reported using zeotropic mixtures, as the working fluid yields the highest overall performance in all three scenarios. The Levelized Cost of Electricity of the proposed systems ranged between 35.42–35.67 EUR/MWh.

In another study, Zhang et al. [37] surveyed the thermodynamic performance of ORC coupled with waste heat recovery from the Rankine cycle, the Brayton cycle, and the thermoelectric generator. R123, R245, and R600 organic fluids in five different operational conditions were examined. The results showed that the DORC is the most efficient configuration, and when employed with the R123, the net output, thermal, and exergy efficiencies were 32.63 kW, 26.55%, and 54.36%, respectively. Thermodynamic and exergoeconomic analyses of ORC in combination with biomass-integrated co-firing and biomass-integrated post-firing technologies—using a mixture of natural gas and biomass as a feedstock, and externally fired technology—which solely runs on biomass, have been conducted by Mahramian et al. [38]. Having examined various fluids such as R141b, R123, n-Pentane, HFE7000, and water, they concluded that R141b yields the highest thermal and exergy efficiencies; however, it is the least favorable choice financially.

Wang et al. [39] proposed a novel organic Rankine cycle-based micro-scale cogeneration system operating with a two-stage pressure evaporator. Given the operational characteristics of the system, they examined R141b and R123 and deduced that the former results in lower capital costs and higher performance. Applying a versatile optimization algorithm, they found that the system is capable of producing 1.66 kW_e_ and 37.16 kW_th_ at the thermal efficiencies of 77.8% and 11.28% for CHP and ORC, respectively. The capital rate is estimated to be 0.363 USD/h. Oyekale et al. [40] studied the techno-economic aspects of biomass retrofitting in hybrid concentrated solar power biomass ORC (CSP-Biomass ORC) power plants in both constant and modular states. They used an operating CSP-ORP facility located at Ottana, Itlay, which includes linear Fresnel collectors coupled with two thermal oil storage tanks and a 630 kW ORC unit, as their case study. The results demonstrated that biomass retrofitting at best leads to 5% electrical efficiency improvement and up to 3500 h increase in operational hours. The payback period of the proposed method is 1.4 years with LCOE and NPV of 109 GBP/MWh and GBP 1.83 million, respectively.

**Figure 1:**
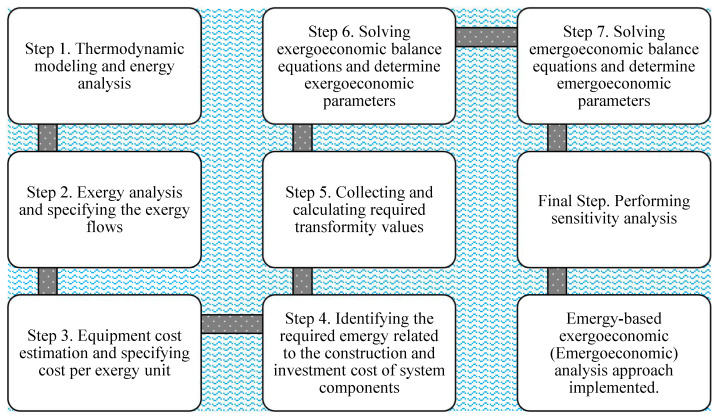
Flowchart of the implemented emergoeconomic analysis.

Reviewing the literature around ORCs demonstrates that numerous researchers have carried out the optimization and performance improvement of ORCs in various applications. In particular, utilizing ORCs to recover waste heat produced in various processes has been extensively studied from thermodynamic, economic, and environmental viewpoints. However, to the best of the authors’ knowledge, an emergy-based exergoeconomic (emergoeconomic) study of biomass combustion heat recovery combined with an ORC has not yet been carried out. Hence, to fill this knowledge gap, a comprehensive evaluation of a BCWHR-ORC from the energy, exergy, exergoeconomic, and emergoeconomic perspectives have been conducted in this study. The emergoeconomic approach, first introduced by Aghbashlo and Rosen [41], is one of the most robust methods used for analyzing energy systems in recent years. The results not only contain comprehensive information on thermoeconomic characteristics but also provide an appropriate understanding of a system’s stability. The mentioned approach is adopted in this study through the eight steps shown in the Figure 1 flowchart. In the next sections, the following is discussed: Section 2 provides a descriptive account of the proposed system. In Section 3, governing equations and the math behind the modeling are discussed thoroughly, while Section 4 presents the modeling outputs and the results of a sensitivity analysis to account for parameter uncertainties. Finally, the paper is concluded in Section 5.

## 2. System Description

**Figure 2:**
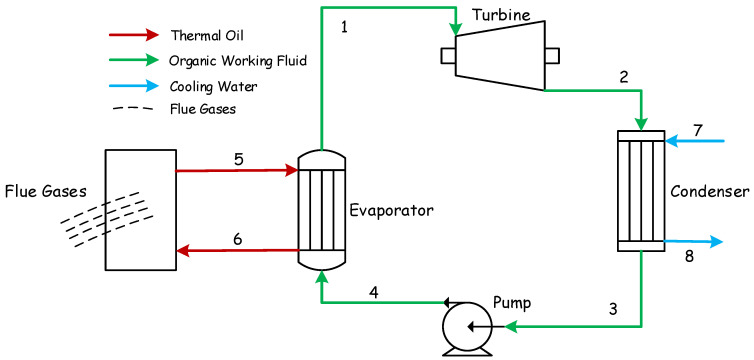
Schematic of the BCWHR-ORC system.

**Figure 3:**
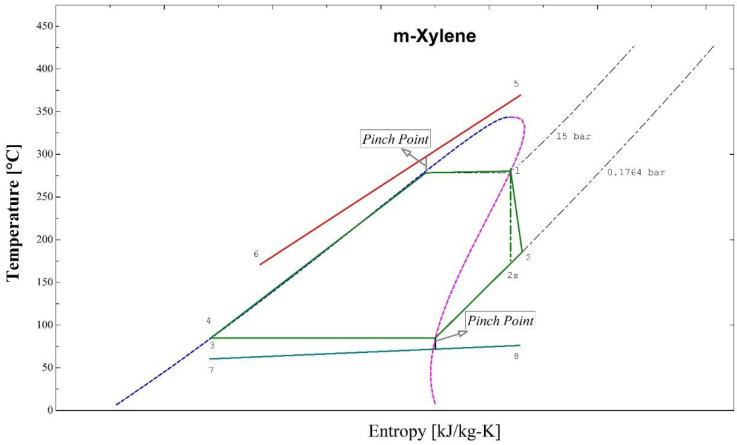
T-S (Temperature-Entropy) diagram of the BCWHR-ORC system.

Figure 2 shows the BCWHR-ORC system studied in this paper. Evaporation takes place within the evaporator, in which the heat is transferred from thermal oil to the organic working fluid at a constant pressure. The thermal oil heats the working fluid from the subcooled liquid phase (point 4) at the pump’s output to the superheated steam required for the turbine operation (point 1). Figure 3 depicts the whole process in the T-S diagram. As the organic fluid passes through the turbine, it expands, generates power by rotating the turbine’s shaft, and builds its pressure up to the working pressure of the condenser (point 2); (note that point 2s in the diagram represents the isentropic expansion process within the turbine). Then, the working fluid is cooled down at the condenser’s pressure by expelling its heat into the cooling water cycle (7→8). Finally, the saturated liquid (point 3) is compressed up to the evaporator’s pressure (point 4), and the process repeats. The thermal oil gains its heat content from the flue gas of a biomass incinerator, enters the evaporator at a high temperature (point 5), and exits at a lower temperature (point 6) after losing its heat to the ORC fluid.

### Selection of the Working and Heat Transfer Fluids

Generally, flue gas heat can be recovered either directly or indirectly. In the direct method, the heat is exchanged between the organic working fluid and flue gas through a heat exchanger. However, the indirect method uses a thermal oil loop as a heat transfer medium. The latter has been studied in this paper to avoid working fluid disintegration due to the high flue gas temperature. To select a suitable thermal oil, characteristics such as having high thermal stability, conductivity, and heat capacity should be considered. On the other hand, a low expansion coefficient and viscosity—to minimize the volumetric change and the compression work—low inflammability, and being non-toxic are the other determinants for choosing a thermal oil. Consequently, Therminol VP-1 was used in this system as the thermal oil, the complete specifications of which can be found in reference [42].

Organic fluids with a high critical temperature provide better thermodynamic performance. Lai et al. [43] have suggested linear siloxanes, alkanes, and aromatic compounds suitable for high-temperature ORC systems. In this study, m-xylene, which was previously studied in [44], and water are chosen as the working fluid and the coolant, respectively.

## 3. Mathematical Modeling

The BCWHR-ORC system mentioned above has been modeled using EES software. The following assumptions were adapted to simplify the developed code:

1. Steady-state operation.
2. Negligible pressure loss in the condenser, the evaporator, and the piping.
3. Negligible heat loss through equipment.
4. Atmospheric pressure and room temperature (298 K) are assumed as the dead state for exergy calculation.

The static parameters introduced in the model are also shown in Table 1.

### 3.1. First Law of Thermodynamics (Energy Concept)

The mass and energy conservation equations (Equations (1) and (2)), excluding potential and kinetic energy terms, were used to study the system equipment.

(1) $$\sum {\dot{m}}_{i}=\sum {\dot{m}}_{e}$$

(2) $$\sum \dot{Q}+\sum {\dot{m}}_{i}h_{i}=\sum \dot{W}+\sum {\dot{m}}_{e}h_{e}$$

Table 2 represents the previous equations, rewritten for each piece of equipment. The thermal efficiency equation of the whole system is given below:

(3) $$\eta_{th}=\frac{{\dot{W}}_{net}}{{\dot{Q}}_{in}}$$

(4) $${\dot{W}}_{net}={\dot{W}}_{T}-{\dot{W}}_{P}$$

The total heat transfer coefficient (*UA*) of the systems’ heat exchangers (condenser and evaporator) is calculated using the following equations:

(5) $$UA_{total}=\frac{{\dot{Q}}_{Evap}}{\Delta T_{LMTD,Evap}}+\frac{{\dot{Q}}_{Cond}}{\Delta T_{LMTD,Cond}}$$

(6) $$\Delta T_{LMTD,Evap}=\frac{\left(T_{5}-T_{1}\right)-\left(T_{6}-T_{4}\right)}{ln\frac{\left(T_{5}-T_{1}\right)}{\left(T_{6}-T_{4}\right)}}$$

(7) $$\Delta T_{LMTD,Cond}=\frac{\left(T_{2}-T_{8}\right)-\left(T_{3}-T_{7}\right)}{ln\frac{\left(T_{2}-T_{8}\right)}{\left(T_{3}-T_{7}\right)}}$$

### 3.2. Second Law of Thermodynamics (Exergy Concept)

To assess the performance of a system, particularly from a financial perspective, exergy analysis is a viable method for determining exergy loss and its destruction rates in the system equipment to identify potential performance improvement possibilities. Given that no chemical reaction occurs within the process and that there are negligible contributions from kinetics and potential energies, the exergy flow rate at a certain point in the system is expressed as follows:

(8) $${\dot{Ex}}_{total,i}={\dot{Ex}}_{ph,i}=\dot{m}\left[\left(h_{i}-h_{o}\right)-T_{0}\left(s_{i}-s_{o}\right)\right]$$

where $h_{o}$ and $s_{o}$ are the enthalpy and entropy values at the dead state, respectively. Ignoring heat loss to the surrounding environment, the rate of exergy destruction for each piece of equipment is calculated using Equation (9):

(9) $${\dot{Ex}}_{D,k}={\dot{Ex}}_{F,k}-{\dot{Ex}}_{P,k}$$

Additionally, the ratio of exergy destruction, which calculates the share of each piece of equipment in the total exergy destruction, is given below:

(10) $$y_{D,k}=\frac{{\dot{Ex}}_{D,k}}{{\dot{Ex}}_{D,total}}$$

where

(11) $${\dot{Ex}}_{D,total}={\dot{Ex}}_{D,Evap}+{\dot{Ex}}_{D,Turb}+{\dot{Ex}}_{D,Cond}+{\dot{Ex}}_{D,Pump}$$

Finally, the efficiency of the second law (exergy efficiency) for each component and the whole system is calculated using Equations (12) and (13), respectively:

(12) $$\varepsilon_{k}=\frac{{\dot{Ex}}_{P,k}}{{\dot{Ex}}_{F,k}}\times 100=\left(1-\frac{{\dot{Ex}}_{D,k}}{{\dot{Ex}}_{F,k}}\right)\times 100$$

(13) $$\varepsilon_{total}=\frac{{\dot{Ex}}_{P,total}}{{\dot{Ex}}_{input}}\times 100=\left(\frac{{\dot{W}}_{net}}{{\dot{Ex}}_{5}-{\dot{Ex}}_{4}}\right)\times 100=\left(\frac{{\dot{W}}_{T}-{\dot{W}}_{P}}{{\dot{m}}_{oil}\times \left(ex_{5}-ex_{4}\right)}\right)\times 100$$

The exergy efficiency and the destruction rate equation of the system components are included in Table 3.

### 3.3. Exergoeconomic Analysis

Exergoeconomics is an engineering tool that combines exergy and financial analyses to achieve cost-effective system design. Through an exergoeconomic analysis, the efficacy of energy conversion systems is compared, concerning the unit cost of products. Such a comparison is not feasible when conducting separate exergy and financial analyses. Various methods, including the cost of energy theory [45], the average cost approach [46], and the specific exergy cost (SPECO) theory [47], have been proposed for exergoeconomic system analyses. The latter, which consists of the following three steps, has been applied in this study:

1. Determining energy and exergy flows at the component’s boundaries.
2. Determining the fuel and product of each component.
3. Developing cost balance and auxiliary equations for the system components using Equation (14):
  (14) $$\underset{e}{\sum }{\dot{C}}_{e,k}+{\dot{C}}_{W,k}=\underset{i}{\sum }{\dot{C}}_{i,k}+{\dot{C}}_{Q,k}+{\dot{Z}}_{k}$$
  where ${\dot{C}}_{j}=c_{j}\dot{E_{j}}$, so Equation (11) is rewritten as Equation (15):
  (15) $$\underset{e}{\sum }{\left(c_{e}{\dot{Ex}}_{e}\right)}_{k}+{\left(c_{w}\dot{W}\right)}_{k}=\underset{i}{\sum }{\left(c_{i}{\dot{Ex}}_{i}\right)}_{k}+{\left(c_{q}{\dot{Ex}}_{q}\right)}_{k}+{\dot{Z}}_{k}$$
  where $\;c_{i}$*,* $c_{e}$*,* $c_{q}$ and $c_{w}$ represent the cost per unit of exergy in USD/GJ.$\;{\dot{Z}}_{k}\;$ is the total investment cost of the *k*_th_ component, including the capital cost rate $\left({\dot{Z}}_{k}^{CI}\right)$ and the O&M cost (${\dot{Z}}_{k}^{OM}$). To calculate ${\dot{Z}}_{k}$, certain parameters such as the capital return factor (CRF), O&M factor ($\varphi_{r}$), annual operating hours (OH), and capital cost of the *k*_th_ equipment $\left(PEC_{k}\right)$, are to be defined. Finally, ${\dot{Z}}_{k}$ is calculated for all components using Equations (16) and (17) [48]:
  (16) $${\dot{Z}}_{k}={\dot{Z}}_{k}^{CI}+{\dot{Z}}_{k}^{OM}=\frac{\left(PEC_{k}\times CRF\times \varphi_{r}\right)}{OH\times 3600}$$
  (17) $$CRF=\frac{i{\left(i+1\right)}^{n}}{{\left(i+1\right)}^{n}-1}$$
  where *i* and *n* are the discount ratio and the system’s lifetime, respectively. In this study, the bare module cost method was adopted to calculate the equipment purchase cost of the system [49,50]. Equations (18)–(25) are represent the capital cost calculation of the heat exchangers (evaporator and condenser), the turbine, and the pump, respectively:
  (18) $$PEC_{HE}=C_{0,HE}\times \left[B_{1,HE}+\left(B_{2,HE}\times F_{M,HE}\times F_{P,HE}\right)\right]$$
  (19) $$logC_{0,HE}=\left[K_{1,HE}+K_{2,HE}\left(logA_{HE}\right)+K_{3,HE}{\left(logA_{HE}\right)}^{2}\right]$$
  (20) $$logF_{P,HE}=\left[C_{1,HE}+C_{2,HE}\left(logP_{HE}\right)+C_{3,HE}{\left(logP_{HE}\right)}^{2}\right]$$
  (21) $$PEC_{Turbine}=C_{0,T}\times F_{M,T}$$
  (22) $$logC_{0,T}=\left[K_{1,T}+K_{2,T}\left(log{\dot{W}}_{T}\right)+K_{3,T}{\left(log{\dot{W}}_{T}\right)}^{2}\right]$$
  (23) $$PEC_{Pump}=C_{0,P}\times \left[B_{1,P}+\left(B_{2,P}\times F_{M,P}\times F_{P,P}\right)\right]$$
  (24) $$logC_{0,P}=\left[K_{1,P}+K_{2,P}\left(log{\dot{W}}_{P}\right)+K_{3,P}{\left(log{\dot{W}}_{P}\right)}^{2}\right]$$
  (25) $$logF_{P,P}=\left[C_{1,P}+C_{2,P}\left(log_{10}P_{P}\right)+C_{3,P}{\left(log_{10}P_{P}\right)}^{2}\right]$$

In the above equations,$\;C_{0}$, $F_{M}$, $F_{P}$, A, and P are the initial cost, material factor, pressure factor, surface area, and equipment pressure, respectively. Additionally,$\;B_{1}$, $B_{2}$, $C_{1}$, $C_{2}$, $C_{3}$, $K_{1}$, $K_{2}$, and $K_{3}$ are material constants—their values are given in Table 4. In this study, carbon steel is assumed to be the equipment fabrication material due to its high durability against water and the organic working fluid. Note that the calculated equipment initial costs via Equations (18)–(25) are based on 2001 data as the reference year. Using Equation (26) and the chemical engineering plant cost index (*CEPCI*), the costs can be projected to its equivalent values in 2019:

(26) $$PEC_{k,2019}=\frac{CEPCI_{2019}}{CEPCI_{2001}}\times PEC_{k,2001}$$

After calculating the components’ initial costs using their relevant auxiliary equations derived from the F and P principles of the SPECO method [47], the cost balance equations of all components are solved within the EES simultaneously. The cost balance and auxiliary equations for the system components are provided in Table 5. Additionally, the static parameters employed for the exergoeconomic analysis are listed in Table 6.

To better understand the exergoeconomic performance of individual components, it is necessary to define the key parameters of the analysis. These include average cost per unit exergy of fuel ($c_{F,k}$) and product ($c_{P,k}$), cost flow rate of exergy destruction (${\dot{C}}_{D,k}$), total cost rate (${\dot{C}}_{Tot,k}$), relative cost difference ($r_{k}$), and the exergoeconomic factor ($f_{k}$), which are defined respectively in Equations (27)–(31) [48] (losses have not been included):

(27) $$c_{F,k}=\frac{{\dot{C}}_{F,k}}{{\dot{Ex}}_{F,k}}\;,\;\;c_{P,k}=\frac{{\dot{C}}_{P,k}}{{\dot{Ex}}_{P,k}}$$

(28) $${\dot{C}}_{D,k}={\dot{C}}_{F,k}{\dot{Ex}}_{D,k}$$

(29) $${\dot{C}}_{Tot,k}={\dot{Z}}_{k}+{\dot{C}}_{D,k}$$

(30) $$r_{k}=\frac{c_{P,k}-c_{F,k}}{c_{F,k}}$$

(31) $$f_{k}=\frac{{\dot{Z}}_{k}}{{\dot{Z}}_{k}+{\dot{C}}_{D,k}}$$

In Equation (31), $r_{k}$ represents the difference between the specific cost of fuel and the product for a given component due to the existing cost rates of exergy destruction ${\dot{C}}_{D,k}$ and capital investment ${\dot{Z}}_{k}$. The exergoeconomic factor $f_{k}\;$in Equation (28) quantifies the relative importance of a component’s cost to its exergy destruction cost [48].

### 3.4. Emergy Concept

Emergy refers to the amount of available energy required, either directly or indirectly, to generate a given output flow or storage of energy or service, expressed as solar equivalent joules (sej). The concept was developed in the 80s by Odum et al. at the University of Florida, based on the thermodynamic principle and general systems theory to evaluate their long-term sustainability [54]. Emergy is a suitable measure for calculating the consumption of natural resources as a function of equivalent solar energy [55]. In this method, a transformity coefficient or Unit Emergy Value (UEV) is defined to convert all process inputs such as energy, materials, workforce, and financial services to a type of available energy, for instance, solar equivalent joules (sej). The transformity values are emergy-based energy; therefore, to conduct an emergoeconomic analysis, these values should be multiplied by the scale factor (β=0.93) presented in [56] to result in the respective emergy-based exergy values. This factor is the exergetic equivalent of solar energy and represents the maximum extractable work from a solar energy flow, calculated through Equation (32):

(32) $$\beta =1+\frac{1}{3}{\left(\frac{T_{0}}{T_{S}}\right)}^{4}-\frac{4}{3}\left(\frac{T_{0}}{T_{S}}\right)$$

where $T_{0}\;$and $T_{S}\;$are the base temperature and solar temperature, respectively. 

In this study, the flue gas of the biomass combustor is the driving force of the system, so the determination of the amount of emergy it enters into the system is required. Due to the lack of data on the transformity coefficient of biomass combustion waste heat, the reported Equation (33) for the transformity ratio of coal-fired waste heat by Zhang et al. [57] is employed in this study:

(33) $${Transformity}_{WH}=\frac{Tr_{biomass}\times \Delta T}{\eta_{biomass}\times T}$$

In Equation (33), $Tr_{biomass}$ is the transformity of the output power of a conventional biomass power plant, expressed as (sej/J), ΔT is the temperature difference of the working fluid in the evaporator and the condenser in terms of K, $\eta_{biomass}$ is the ideal (Carnot) efficiency of the power plant, and T is the working fluid temperature in the evaporator (K). The $Tr_{biomass}$ and $\eta_{biomass}$ values are determined from [58,59].

### 3.5. The Emergy-Based Exergoeconomic Analysis (Emergoeconomic)

The emergoeconomic analysis method introduced by Aghbashlo and Rosen [41] is a practical and powerful tool for measuring the long-term sustainability of energy systems. This method has similar rules to exergoeconomic analyses. The SPECO method is calculates the monetary emergy values of all exergy flows. To this end, first, a monetary emergy rate (${\dot{M}}_{i}$) was defined by the following:

(34) $${\dot{M}}_{i}=m_{i}{\dot{Ex}}_{i}$$

where $m_{i}$ denotes the monetary emergy per exergy unit (specific monetary emergy) in terms of sej/exergy unit, and ${\dot{M}}_{i}\;$is the rate of monetary emergy in terms of sej/time unit. An emergoeconomic balance equation for a given component is written as:

(35) $${\dot{M}}_{P,k}={\dot{M}}_{F,k}+{\dot{U}}_{k}$$

where ${\dot{M}}_{P,k}$ and ${\dot{M}}_{F,k}$, respectively, denote the rate of monetary emergy for product and fuel flows. The emergoeconomic equations of the system components are provided in Table 7. Through Equations (36) and (37), the specific monetary emergy of both the product and the fuel is calculated:

(36) $$m_{F,k}=\frac{{\dot{M}}_{F,k}}{{\dot{Ex}}_{F,k}}$$

(37) $$m_{P,k}=\frac{{\dot{M}}_{P,k}}{\dot{E}x_{P,k}}$$

Accordingly, the monetary emergy rate of exergy destruction is obtained from Equation (38):

(38) $${\dot{M}}_{D,k}=m_{F,k}{\dot{E}}_{D,k}$$

${\dot{U}}_{k}$ is the investment-related monetary emergy rate for each component given as follows:

(39) $${\dot{U}}_{k}={\dot{U}}_{k}^{CI}+{\dot{U}}_{k}^{OM}=\frac{\left(EM_{k}\times CRF\times \varphi_{r}\right)}{N\times 3600}$$

In the above equation, ${\dot{U}}_{k}^{CI}$ and ${\dot{U}}_{k}^{OM}$ denote the rate of monetary emergy of capital investment and the O&M, respectively. $EM_{k}$ represents the emergy content of the $k_{th}$ component and is obtained using Equations (40)–(42):

(40) $$EM_{k}=EM_{construct,k}+EM_{Purchase,k}$$

(41) $$EM_{construct,k}=Mass_{k}\times EM_{material}$$

(42) $$EM_{Purchase,k}=PEC_{k}\times EM_{dollar}$$

where $EM_{construct,k}$, and $EM_{Purchase,k}$, are the emergy content relevant to the construction and purchasing of the $k_{th}$ component in sej, ${Mass}_{k}$ is its respective mass in grams, $EM_{material}$ is the emergy of the construction material (sej/gr), and $EM_{dollar}$ is the monetary emergy in sej/USD. Note that all emergy values are based on energy, so they have to be multiplied by β=0.93 to obtain their corresponding exergy-based emergy values. 

The total emergy rate of a given component is obtained through the summation of the monetary emergy rates related to both the investment (${\dot{U}}_{k})\;$and the exergy destruction (${\dot{M}}_{D,k})$ using Equation (43):

(43) $${\dot{M}}_{Tot,k}={\dot{U}}_{k}+{\dot{M}}_{D,k}$$

The relative monetary emergy between corresponding average values of the product and the fuel is calculated using the following equation:

(44) $$r_{m,k}=\frac{m_{P,k}-m_{F,k}}{m_{F,k}}\times 100$$

Additionally, Equation (45) is used to calculate the emergoeconomic factor of each system component:

(45) $$F_{m,k}=\frac{{\dot{U}}_{k}}{{\dot{U}}_{k}+{\dot{M}}_{D,k}}\times 100=\frac{{\dot{U}}_{k}}{{\dot{M}}_{Tot,k}}\times 100$$

## 4. Results and Discussion

### 4.1. Verification

To validate the developed code, the results derived from modeling the BCWHR-ORC system in this study were compared with those presented in [60], which closely resembles our case study. Two performance indicators, namely, the maximum power output and the maximum thermal efficiency, were compared at five distinct waste heat source temperatures ranging from 325 °C to 365 °C, over three different pinch point temperatures (5/5, 10/10, and 5/10) and varying evaporator pressure from 11 to 36 bar. As shown in Table 8, the results are in good agreement with [60].

### 4.2. The Energy Analysis Results

In this section, the mass and energy balance equations for the components were developed using the first law of thermodynamics (Table 2), and then the thermodynamic characteristics at various system points were calculated, as shown in Table 9. As mentioned previously, the optimal BCWHR-ORC studied in [46] was chosen as the case study. The system’s net power and the total heat transfer surface area were 160 kW and 16.18 kW/K, respectively.

### 4.3. The Exergy Analysis Results

**Figure 4:**
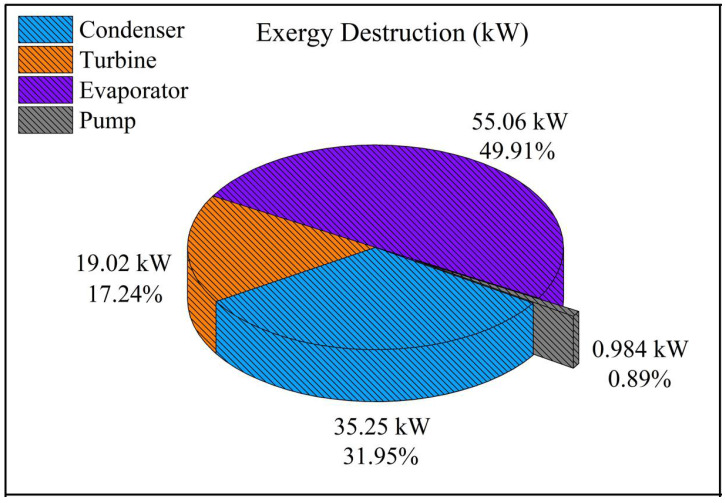
The rate of exergy destruction attributed to each component.

**Figure 5:**
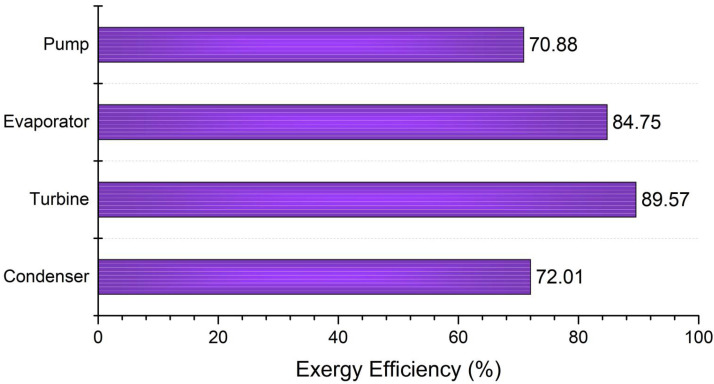
The exergy efficiency of the system equipment.

To investigate the exergy performance of an energy system, all exergy flows in the system’s components should be determined. Subsequently, the rate of exergy destruction and the exergy efficiency for each system component were obtained by applying the second law of thermodynamics (Table 3). Figure 4 depicts the exergy destruction rate for the equipment. As can be seen, the highest values were attributed to the evaporator, condenser, turbine, and pump, respectively. The evaporator accounted for about 50% of the total exergy destruction rate, owing to the heat transfer at a large temperature difference between the thermal oil and the working fluid. Likewise, the exergy destruction of the condenser was 31.95% of the total value, which is due to the high temperature of the cooling water resulting in a significant exergy flow out of the system. To remedy this and to improve the overall system performance, the cooling water leaving the condenser should be utilized as a high-temperature heat source for a downstream process, as proposed by Aziz et al. [60]. The exergy efficiency of the system equipment is shown in Figure 5, in which the turbine, evaporator, condenser, and pump, at 89.57%, 84.75%, 72.01%, and 70.88%, have the highest efficiency, respectively.

### 4.4. The Exergoeconomic Analysis Results

**Figure 6:**
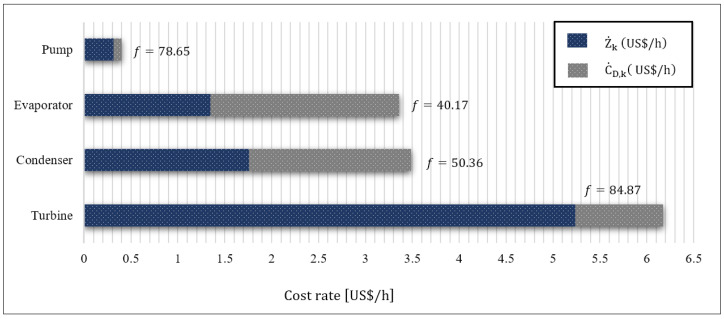
The bar diagram of the investment (${\dot{Z}}_{k}$) cost rate and the exergy destruction cost rate (${\dot{C}}_{D,k}$) cost rates of the system equipment.

To perform the exergoeconomic analysis, first the capital investments required for the equipment purchase were calculated using Equations (18)–(25) and the Table 4 data. Then, to determine cost flows in and out of the components, the corresponding cost balance equations were written, based on Table 5. These equations, along with auxiliary equations compliant with F and P principles and the biomass combustion equation, were solved to calculate the cost of flue gas streamed into the process. The values of the exergy flow, cost rate per exergy unit, and cost rate of the BCWHR-ORC system flow are provided in Table 10. Additionally, Table 11 summarizes the values of the component parameters obtained in the exergoeconomic analysis. Meanwhile, Figure 6 compares the rates of investment cost and the exergy destruction for each piece of equipment. According to Table 10, the cost rate of the output power and the cost rate of the output power per exergy unit were 14.19 USD/h and 24.13 USD/GJ, respectively. Additionally, the cost rate and cost rate per exergy unit calculated for the system were 52.45 USD/h, and 91.47 USD/GJ, respectively.

Based on Table 11, the turbine with 5.238 USD/h had the highest investment cost rate, followed by the evaporator and the pump. ${\dot{C}}_{D},$ representing the cost rate of exergy destruction, was the highest for both the evaporator and the condenser, with the former leading. This denotes that both components had the highest destruction rate from an exergoeconomic perspective, thus demanding further study to apply measures to reduce their respective cost rates. In contrast, the turbine and pump, with the exergy destruction cost rate of 0.9335 USD/h and 0.0855 USD/h, respectively, indicate a lower potential for improvements in their performance characteristics to reduce their exergy destruction cost rates. To ease the comparison, Figure 6 depicts the ${\dot{Z}}_{k}\;$and ${\dot{C}}_{D,k}\;$of each component. From an exergoeconomic point of view, the higher the total cost rate ${\dot{C}}_{Total}$ for a given component in a system, the more that component should be considered for the evaluation (both in terms of capital cost and the exergy destruction) of performance improvement opportunities. As a result, the turbine, with the highest ${\dot{C}}_{Total}$, is of interest for performance improvement in this study. A high ${\dot{C}}_{Total}$ for a component could be either due to the high capital cost or the high exergy destruction cost rate; for the turbine, the former is the case. Following the turbine, the condenser and the evaporator, with 3.4862 USD/h and 3.3592 USD/h, respectively, had the highest ${\dot{C}}_{Total}$ in the system, indicating their suitable exergoeconomic potentials for performance improvement.

A high exergoeconomic factor (*f*) for a component emphasizes the significance of its investment cost rate against its exergy destruction cost rate. As shown in Table 11, the turbine, at 84.87%, had the highest f factor value. This implies that the turbine’s capital cost composed the majority of its cost rates, and from an exergoeconomic perspective, it has to be reduced for performance improvement. The pump, the condenser, and the evaporator are prioritized next for the evaluation of capital investment cost reduction. The low *f* factor of the evaporator indicates the prevalence of the exergy destruction cost rate in this piece of equipment. Additionally, a high exergy destruction cost rate $\left({\dot{C}}_{D}\right)$ in a component results in a correspondingly high relative cost difference (r). As it is evident from Table 10, the pump had the highest r value, indicating the significance of its cost rate of exergy destruction over its capital investment cost. The majority of exergy destruction in the pump is attributed to its low efficiency. Replacing the existing pump with an efficient one reduces both the rate of exergy destruction and the r value. Placed the next in priority for reducing the exergy destruction rate, are the condenser, and the turbine, respectively.

### 4.5. The Results of the Emergoeconomic Analysis

The emergoeconomic evaluation of the BCWHR-ORC system was carried out according to the methodology described in Section 3.4. Based on Equation (33), the transformity coefficient of the biomass waste heat was $2.77\times 10^{4}\;\mathrm{sej}/J$. Having determined this coefficient, the emergoeconomic cost balance and the auxiliary equations for the system’s components (Table 7) were solved simultaneously to calculate the values of the monetary emergy per exergy unit and the monetary emergy rate of the equipment. According to Table 12, the monetary emergy per exergy unit of the turbine power was $4.77\times 10^{13}\mathrm{sej}/\mathrm{GJ}$. Since the turbine power is the final output of the system, it is expected to have the highest value among the other system flows.

Table 13 shows the monetary energy values of all the system components. To obtain these values, their respective investment costs should be multiplied by $9.95\times 10^{11}\;\mathrm{sej}/\mathrm{USD}$, which is the ratio of the emergy to the dollar [41]. Consequently, with the highest investment cost, the turbine also had the highest monetary emergy value related to equipment purchasing, equal to $3.12\times 10^{17}\;\mathrm{sej}$.

Additionally, similar to the monetary emergy of equipment’s capital investment, the monetary emergy related to their construction also needs to be calculated. Table 14 provides the energy and exergy-based monetary emergy values of the required materials for the power plant construction. The turbine was the heaviest component, hence requiring more steel for its fabrication. Following the turbine were the condenser, the evaporator, and the pump, also demanding large values of monetary emergy for construction. 

**Figure 7:**
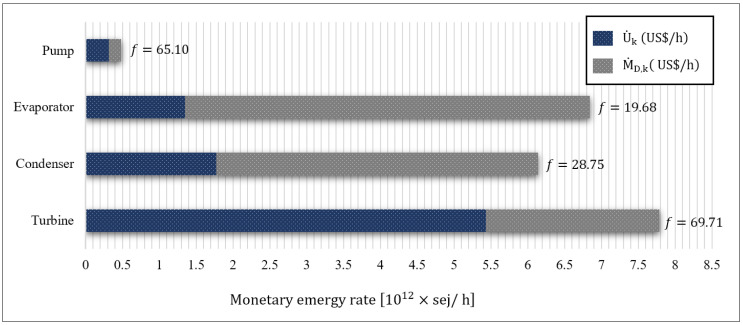
The bar diagram of the monetary emergy rate of capital investment (${\dot{U}}_{k}$) and the monetary emergy rate of exergy destruction (${\dot{M}}_{D,k}$) for the system equipment.

Finally, Table 15 summarizes the output of the emergoeconomic analysis of the system, presented graphically in Figure 7. As expected, owing to the high monetary emergy rate of capital cost (${\dot{U}}_{k}$) and the turbine’s weight, it had the highest monetary emergy rate of investment, with a value of $5.43\times 10^{12}\;\mathrm{sej}/h$. Ranking from the highest to the lowest total monetary emergy rate (${\dot{M}}_{total})\;$were the turbine, the evaporator, the condenser, and the pump, with the respective values of $7.79\times 10^{12}\;\mathrm{sej}/h$, $6.84\times 10^{12}\;\mathrm{sej}/h$, $6.14\times 10^{12}\;\mathrm{sej}/h$, and $4.85\times 10^{11}\;\mathrm{sej}/h$. The high ${\dot{M}}_{Total}$ was attributed to either ${\dot{U}}_{k}$ or ${\dot{M}}_{D,k}$, implicating the ample potential for performance improvement from an emergoeconomic perspective. In the present system, more than 53% of ${\dot{M}}_{Total}$ was due to the exergy destruction occurring in the equipment. As a result, lowering the irreversibility of the equipment’s monetary emergy, particularly in the evaporator and the condenser, improves the overall performance of the system.

The emergoeconomic factor ($f_{m}$) of equipment varied from 19.68% to 69.71%, with the latter belonging to the turbine. This denotes that ${\dot{U}}_{Turb}$ prevails over ${\dot{M}}_{D,Turb}$, meaning that the main contribution to ${\dot{M}}_{D,Turb}$ was from the ${\dot{U}}_{Turb}$ term. Therefore, to lower the $f_{m,Turb}$, one should attempt to decrease its capital investment cost. Contrary to the turbine, the evaporator’s exergoeconomic factor was 19.68%, implicating ${\dot{M}}_{D,Evap}$ dominance over ${\dot{U}}_{Evap}—$ thus requiring the evaporator to be further investigated for lowering the exergy destruction rate. Moreover, increasing the evaporator’s monetary emergy rate of investment due to the utilization of auxiliary equipment to minimize irreversibility is recommended.

The relative monetary emergy difference ($r_{m}$) for the equipment ranged from 22.41% to 117.6%, with the latter attributed to the pump. Following the pump were the condenser, the turbine, and the evaporator, at 54.56%, 38.43%, and 22.41%, respectively. As a result, the pump had the highest potential for a decrease in both the monetary emergy of investment and the monetary emergy of exergy destruction (${\dot{M}}_{Total,Pump}$). On the other hand, the evaporator had the lowest $r_{m}$, indicating the difficulty of improving the equipment’s monetary emergy of the product.

**Figure 8:**
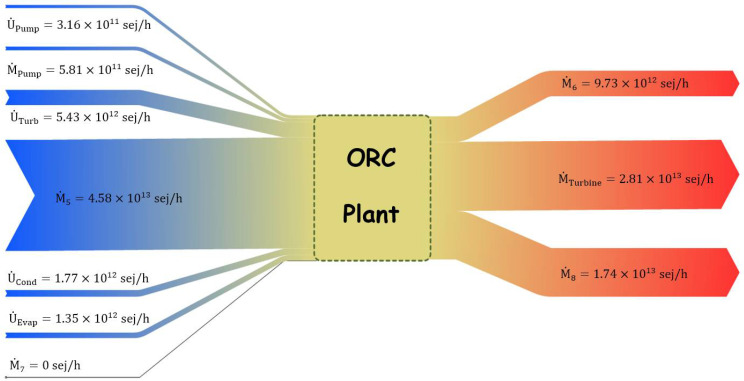
The Grassmann diagram for the total monetary emergy flows of the system.

In Figure 8, the Grassmann diagram illustrates all monetary emergy flows entering (left) and leaving (right) the system. The monetary emergy rate of flue gas waste heat (${\dot{M}}_{5}$), as the driving force of the system, was the highest amongst all the other flows, with the value of $4.58\times 10^{13}\;\mathrm{sej}/h$. The monetary emergy rate of the cooling water (${\dot{M}}_{7}$) was obtained at zero (according to the auxiliary emergoeconomic equations), and the remaining flows were attributed to ${\dot{U}}_{k}$. Meanwhile, three primary flows left the power plant, between which the turbine’s net power had the highest monetary emergy rate, equal to $2.81\times 10^{13}\;\mathrm{sej}/h$.

### 4.6. The Sensitivity Analysis

While applying the emergoeconomic analysis, it may not be possible to accurately obtain the transformity coefficient for some processes owing to their complex and time-consuming calculations. Sometimes, the transformity coefficient of a given process is estimated using a correlation, such as Equation (33), used in this paper for calculating the BCWH transformity coefficient. Consequently, it is necessary to determine the uncertainty of the calculated monetary emergy values of the system due to the estimated transformity coefficient. For this purpose, the ψ parameter is defined to quantify the overall emergoeconomic performance of the system:

(46) $$\psi =\frac{{\dot{M}}_{Turbine}+{\dot{M}}_{8}}{{\dot{M}}_{5}+{\dot{M}}_{7}+{\dot{M}}_{Pump}+{\dot{U}}_{Evap}+{\dot{U}}_{Turb}+{\dot{U}}_{Cond}+{\dot{U}}_{Pump}}\times 100$$

**Figure 9:**
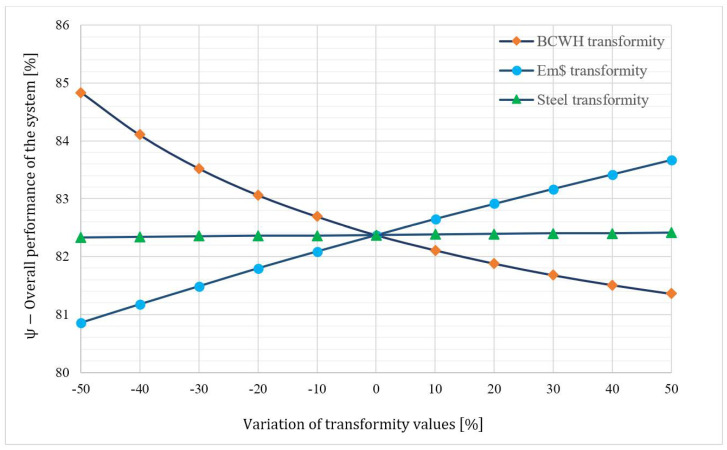
The trend of overall emergoeconomic performance of the system (ψ), with respect to the transformity coefficients.

Figure 9 depicts the trends of$\;Tr_{BCWH}$, $Tr_{emdollar}$, and $Tr_{Steel}$ while changing their values ±50% with 10% increments. As can be seen, ψ was the most sensitive and was inversely correlated to $Tr_{BCWH}$ changes, which is the main driving force of the system. The higher the emergy content of BCWH is, the lower the ψ value will be. However, it should be noted that the changes in ψ were less pronounced compared to ${Tr}_{BCWH}$, as a −50% change in the latter resulted in only a 3% percent decrease in the former. Contrastingly, the $Tr_{emdollar}$, and $Tr_{Steel}$ had a direct correlation with ψ. $Tr_{Steel}$ had hardly any influence on ψ, as it was roughly a straight line drawn parallel to the x-axis. This was because the monetary emergy of the equipment’s construction partook of the least from the total monetary emergy content. $Tr_{emdollar}$ changes also impacted the ψ, since the capital investment constituted the principal share of the equipment’s total emergy content. The ±50% change in $Tr_{emdollar}$, resulted in −1.83% to 1.58% changes in the ψ value.

## 5. Conclusions

In this paper, a BCWHR-ORC system is presented and evaluated from the thermodynamic, economic, and sustainability aspects by implementing energy, exergy, exergoeconomic, and emergoeconomic analyses. Then, a sensitivity analysis was conducted to account for the effect of the transformity coefficient uncertainties on the overall emergoconomic performance of the system. The key results are concluded as follows:

1. The highest rate of exergy destruction (${\dot{Ex}}_{d}$) occurred in the evaporator and the condenser, with the values of 49.91% and 31.95%, respectively. Meanwhile, the turbine, at 89.57%, had the highest exergy efficiency (ε) within the system.
2. According to the exergoeconomic analysis, the cost per exergy unit of the turbine’s power (c) was equal to 24.13 USD/GJ, and its cost rate of output power ($\dot{C}$) was 14.19 USD/h. Additionally, $\dot{C}$ and c for the whole system were 52.45 USD/h and 91.47 USD/GJ, respectively.
3. Amongst the system equipment, the turbine had the highest total cost rate (${\dot{C}}_{Total}$) and exergoeconomic factors (f*)*, with values of 6.1715 USD/h and 84.87%, respectively. It has been deduced that the cost rate of investment for the turbine (${\dot{Z}}_{Turb}$) is large, and measures should be taken to reduce its capital investment cost.
4. Conducting an emergoeconomic analysis, the monetary emergy per exergy unit (m) and the the monetary emergy rate of the output power ($\dot{M}$) were $8.24\times 10^{4}\;\mathrm{sej}/J$, and $4.87\times 10^{13}\;\mathrm{sej}/h$, respectively.
5. The highest monetary emergy rate of capital investment ($\dot{U}$) belonged to the turbine, the condenser, the evaporator, and the condenser, with the corresponding values of $1.77\times 10^{12}$, $5.43\times 10^{12}$, $1.35\times 10^{12}$, and $3.16\times 10^{11}$. In addition, ranked from the highest to the lowest, the monetary emergy of exergy destruction (${\dot{M}}_{D}$) for the evaporator, the condenser, the turbine, and the pumps were $2.64\times 10^{12}$, $4.90\times 10^{12}$, $6.18\times 10^{12}$, and $1.85\times 10^{11}$, respectively.
6. The overall emergoeconomic factor of the BCWHR-ORC was 44.41%, implying that 55.59% of the total monetary emergy was due to exergy destruction (irreversibility) within the system. The reduction of exergy destruction results in system sustainability and performance improvement from an emergoeconomic perspective.
7. The turbine and the pump had the largest emergoeconomic factor ($f_{m,Turb}=67.28$) and relative monetary emergy difference ($r_{m,Pump}=110.8$), respectively, meaning that ${\dot{U}}_{Turb}$ was more pronounced than the ${\dot{M}}_{D,Turb}$; however, for the pump, it was the other way around (${\dot{M}}_{D,Pump}$ dominates ${\dot{U}}_{Pump}$).
8. Using the sensitivity analysis, it was found that the overall emergoeconomic performance of the system (ψ) was the most sensitive to transformity coefficients of the biomass combustion waste heat ($Tr_{BCWH}$) and the emergy per dollar ($Tr_{emdollar}$), respectively.

## Figures and Tables

**Table 1:** Design parameters used in the model [44].

Input Parameter	Unit	Input Value
Thermal power input	kW	1000
Thermal oil inlet temperature	℃	370
Cooling water inlet temperature	℃	70
Flue gases inlet temperature	℃	450
Flue gases exit temperature	℃	180
Condensation Temperature	℃	85
Turbine isentropic efficiency	%	85
Pump isentropic efficiency	%	65
Heating process efficiency	%	85
Evaporator efficiency	%	96
Condenser efficiency	%	98

**Table 2:** Mass and energy balance equations of the system equipment.

Component	Balance Equation
Evaporator	${\dot{Q}}_{Evap}={\dot{m}}_{wf}\left(h_{1}-h_{4}\right)={\dot{m}}_{oil}\left(h_{5}-h_{6}\right)={\dot{m}}_{oil}C_{p_{s}}\left(T_{5}-T_{6}\right)$
Turbine	${\dot{W}}_{T}={\dot{m}}_{wf}\left(h_{1}-h_{2}\right)={\dot{m}}_{wf}\left(h_{1}-h_{2s}\right)\eta_{T}$
Condenser	${\dot{Q}}_{Cond}={\dot{m}}_{wf}\left(h_{2}-h_{3}\right)={\dot{m}}_{cw}\left(h_{8}-h_{7}\right)={\dot{m}}_{cw}C_{p_{cw}}\left(T_{8}-T_{7}\right)$
Pump	${\dot{W}}_{P}={\dot{m}}_{wf}\left(h_{4}-h_{3}\right)={\dot{m}}_{wf}\left(h_{4s}-h_{3}\right)/\eta_{P}$

**Table 3:** The exergy destruction rate and efficiency equations of the system components.

Component	Exergy Balance	Exergy Efficiency
Evaporator	${\dot{Ex}}_{D,Evap}=\left({\dot{Ex}}_{4}-{\dot{Ex}}_{1}\right)+\left({\dot{Ex}}_{5}-{\dot{Ex}}_{6}\right)$	$\left({\dot{Ex}}_{1}-{\dot{Ex}}_{4}\right)/\left({\dot{Ex}}_{5}-{\dot{Ex}}_{6}\right)$
Turbine	${\dot{Ex}}_{D,Turb}=\left({\dot{Ex}}_{1}-{\dot{Ex}}_{2}\right)-{\dot{W}}_{T}$	${\dot{W}}_{T}/$ $\left({\dot{Ex}}_{1}-{\dot{Ex}}_{2}\right)$
Condenser	${\dot{Ex}}_{D,Cond}=\left({\dot{Ex}}_{2}-{\dot{Ex}}_{3}\right)+\left({\dot{Ex}}_{7}-{\dot{Ex}}_{8}\right)$	$\left({\dot{Ex}}_{8}-{\dot{Ex}}_{7}\right)/\left({\dot{Ex}}_{2}-{\dot{Ex}}_{3}\right)$
Pump	${\dot{Ex}}_{D,Pump}=\left({\dot{Ex}}_{3}-{\dot{Ex}}_{4}\right)+{\dot{W}}_{P}$	$({\dot{Ex}}_{4}-{\dot{Ex}}_{3})/{\dot{W}}_{P}$

**Table 4:** Constant Values of the equipment’s capital cost equations.

Constants	Equipment		
	Heat Exchangers	Turbine	Pump
$B_{1}$	1.6300	-	1.8900
$B_{2}$	1.6600	-	1.3500
$C_{1}$	0.0388	-	−0.3935
$C_{2}$	−0.1127	-	0.3957
$C_{3}$	0.0818	-	−0.0023
$K_{1}$	4.3247	2.2476	3.3892
$K_{2}$	−0.3030	1.4965	0.0536
$K_{3}$	0.1634	−0.1618	0.1538
$F_{M}$	1.0000	3.5000	1.5000

**Table 5:** Cost balance and auxiliary equations of the system equipment.

Component	Cost Balance Equation	Auxiliary Equations
Evaporator	$c_{4}{\dot{Ex}}_{4}+m_{5}{\dot{Ex}}_{5}+{\dot{Z}}_{Evap}=m_{1}{\dot{Ex}}_{1}+m_{6}{\dot{Ex}}_{6}$	$c_{5}=c_{6}$
Turbine	$c_{1}{\dot{Ex}}_{1}+{\dot{Z}}_{Turb}=c_{2}{\dot{Ex}}_{2}+c_{W_{T}}{\dot{Ex}}_{W_{T}}$	$c_{1}=c_{2}$
Condenser	$c_{2}\dot{E}x_{2}+c_{7}{\dot{Ex}}_{7}+{\dot{Z}}_{Cond}=c_{3}{\dot{Ex}}_{3}+c_{8}{\dot{Ex}}_{8}$	$c_{2}=c_{3}\;;\;c_{7}=0$
Pump	$c_{3}{\dot{Ex}}_{3}+c_{W_{P}}{\dot{Ex}}_{W_{P}}+{\dot{Z}}_{Pump}=c_{4}\dot{E}x_{4}$	$c_{W_{P}}=c_{W_{T}}$

**Table 6:** Input parameters of the exergoeconomic analysis.

Parameter	Unit	Value	Ref.
Interest rate (i)	%	10	[51]
Plant operational hours (OH)	Hours/year	7446	-
Plant total life time (N)	years	20	[51]
Maintenance factor(φ)	%	6	[51]
Chemical engineering plant cost index 2001 (*CEPCL_2001_*)	-	397	[52]
Chemical engineering plant cost index 2019 (*CEPCL_2019_*)	-	607.5	[52]
Overall heat transfer coefficient of the evaporator (U_Evap)	$\mathrm{kW}/m^{2}$ °C	0.6	[53]
Overall heat transfer coefficient of the condenser (U_Cond)	$\mathrm{kW}/m^{2}$ °C	0.5	[53]

**Table 7:** The auxiliary and emergy balance equations of the system components.

Component	Emergy-Based Cost Balance Equations	Auxiliary Equation
Evaporator	$m_{4}{\dot{E}}_{4}+m_{5}{\dot{E}}_{5}+{\dot{U}}_{Evap}=m_{1}{\dot{E}}_{1}+m_{6}{\dot{E}}_{6}$	$m_{5}=m_{6}$
Turbine	$m_{1}{\dot{E}}_{1}+{\dot{U}}_{Turb}=m_{2}{\dot{E}}_{2}+m_{W_{T}}{\dot{E}}_{W_{T}}$	$m_{1}=m_{2}$
Condenser	$m_{2}{\dot{E}}_{2}+m_{7}{\dot{E}}_{7}+{\dot{U}}_{Cond}=m_{3}{\dot{E}}_{3}+m_{8}{\dot{E}}_{8}$	$m_{7}=0,\;\;m_{2}=m_{3}$
Pump	$m_{3}{\dot{E}}_{3}+m_{W_{P}}{\dot{E}}_{W_{P}}+{\dot{U}}_{Pump}=m_{4}{\dot{E}}_{4}$	$m_{W_{P}}=m_{W_{T}}$

**Table 8:** Comparison of energy analysis results obtained from modeling in the present study (yellow highlighted values) with the values reported in reference [60].

Heat Source Temperature [°C]	ΔT_pp_ = 5/5		ΔT_pp_ = 10/10		ΔT_pp_ = 5/10	
	Maximum Work Output W_max_ [kW]	Maximum Cycle Efficiency η_max_ [%]	Maximum Work Output W_max_ [kW]	Maximum Cycle Efficiency η_max_ [%]	Maximum Work Output W_max_ [kW]	Maximum Cycle Efficiency η_max_ [%]
325	127.7 at 11 bar	15.6 at 11 bar	140.6 at 17 bar	17.2 at 17 bar	146.0 at 21 bar	17.8 at 21 bar
	128.7 at 11 bar	15.7 at 11 bar	141.3 at 17 bar	17.3 at 17 bar	146.5 at 21 bar	17.9 at 21 bar
335	127.7 at 11 bar	15.6 at 11 bar	144.8 at 20 bar	17.7 at 20 bar	150.0 at 25 bar	18.3 at 25 bar
	128.7 at 11 bar	15.7 at 11 bar	145.4 at 20 bar	17.8 at 20 bar	150.4 at 25 bar	18.4 at 25 bar
345	127.7 at 11 bar	15.6 at 11 bar	150.7 at 26 bar	18.5 at 26 bar	152.2 at 28 bar	18.6 at 28 bar
	128.7 at 11 bar	15.7 at 11 bar	151.2 at 26 bar	18.5 at 26 bar	152.6 at 28 bar	18.7 at 28 bar
355	---	---	153.5 at 30 bar	18.8 at 30 bar	154.5 at 32 bar	18.9 at 32 bar
	---	---	153.8 at 30 bar	18.8 at 30 bar	154.9 at 32 bar	19.0 at 32 bar
365	---	---	155.6 at 34 bar	19.1 at 34 bar	156.4 at 36 bar	19.2 at 36 bar
	---	---	155.5 at 34 bar	19.0 at 34 bar	156.5 at 36 bar	19.2 at 36 bar

**Table 9:** Flow type and thermodynamic characteristics at different system points.

State No.	Fluid	Temperature (K)	Pressure (Bar)	Mass Flowrate (kg/s)	Specific Enthalpy (kj/kg)	Specific Entropy (kj/kg K)
1	m-xylene	553.6	15	1.199	777.8	1.714
2	m-xylene	458.8	0.1764	1.199	641.5	1.767
3	m-xylene	358.2	0.1764	1.199	108.5	0.3313
4	m-xylene	359	15	1.199	111.3	0.3341
5	Therminol VP-1	643.2	7.332	1.830	704.3	1.522
6	Therminol VP-1	448.6	7.332	1.830	267.6	0.7188
7	Water	343.2	1	17.63	188.2	0.588
8	Water	351.8	1	17.63	224.5	0.6923

**Table 10:** The exergy rate, cost rate per exergy unit, and the cost rate of system flows.

State No.	Exergy (kW)	*c* (USD/GJ)	$\dot{C}$ (USD/h)
1	320.0	13.64	15.707
2	137.6	13.64	6.7536
3	11.65	13.64	0.5717
4	14.05	23.35	1.1808
5	458.5	10.14	16.736
6	97.50	10.14	3.5590
7	227.8	0	0
8	318.5	6.923	7.9380
Power to Pump	3.384	24.13	6.7536
Turbine Power	163.4	24.13	15.707

**Table 11:** The exergoeconomic outputs of the case study.

Components	${\dot{Z}}_{k}$(USD/h)	${\dot{C}}_{D,k}$(USD/h)	${\dot{C}}_{Tot,k}$(USD/h)	$c_{F,k}$(USD/GJ)	$c_{P,k}$(USD/GJ)	$r_{k}$(%)	$f_{k}$(%)
Evaporator	1.3493	2.0099	3.3592	10.14	13.19	30.08	40.17
Turbine	5.2380	0.9335	6.1715	13.64	24.13	76.96	84.87
Condenser	1.7557	1.7305	3.4862	13.64	24.31	78.31	50.36
Pump	0.3150	0.0855	0.4005	24.13	70.49	192.1	78.65

**Table 12:** The values of the monetary emergy per exergy unit, and the monetary emergy rate of the system flows.

State No.	*m* (sej/GJ)	$\dot{M}$ (sej/GJ)
1	$3.45\times 10^{13}$	$3.97\times 10^{13}$
2	$3.45\times 10^{13}$	$1.71\times 10^{13}$
3	$3.45\times 10^{13}$	$1.45\times 10^{12}$
4	$4.63\times 10^{13}$	$2.34\times 10^{12}$
5	$2.77\times 10^{13}$	$4.58\times 10^{13}$
6	$2.77\times 10^{13}$	$9.73\times 10^{12}$
7	0	0
8	$1.52\times 10^{13}$	$1.74\times 10^{13}$
Power to Pump	$4.77\times 10^{13}$	$5.81\times 10^{11}$
Turbine Power	$4.77\times 10^{13}$	$2.81\times 10^{13}$

**Table 13:** The values of the monetary emergy of the equipment’s capital investment.

Component	Capital Investment Cost (USD)	Energy-Based Transformity (sej/USD)	Exergy-Based Transformity (sej/USD)	Energy-Based Emergy (sej)	Exergy-Based Emergy (sej)
Evaporator	80,699	$1.07\times 10^{12}$	$9.95\times 10^{11}$	$8.64\times 10^{16}$	$8.03\times 10^{16}$
Turbine	313,289	$1.07\times 10^{12}$	$9.95\times 10^{11}$	$3.35\times 10^{17}$	$3.12\times 10^{17}$
Condenser	104,994	$1.07\times 10^{12}$	$9.95\times 10^{11}$	$1.12\times 10^{17}$	$1.05\times 10^{17}$
Pump	18,839	$1.07\times 10^{12}$	$9.95\times 10^{11}$	$2.02\times 10^{16}$	$1.87\times 10^{16}$

**Table 14:** The values of the monetary emergy of the equipment’s construction.

Component	Construction Material	Value	Unit	Energy-Based Transformity (sej/g)	Exergy-Based Transformity (sej/g)	Energy-Based Emergy (sej)	Exergy-Based Emergy (sej)
Evaporator	Steel	$7.43\times 10^{4}$ ^a^	g	$2.77\times 10^{9}$ ^a^	$2.58\times 10^{9}$	$2.06\times 10^{14}$	$1.92\times 10^{14}$
Turbine	Steel	$5.10\times 10^{6}$ ^a^	g	$2.77\times 10^{9}$ ^a^	$2.58\times 10^{9}$	$1.41\times 10^{16}$	$1.31\times 10^{16}$
Condenser	Steel	$4.25\times 10^{5}$ ^a^	g	$2.77\times 10^{9}$ ^a^	$2.58\times 10^{9}$	$1.18\times 10^{15}$	$1.09\times 10^{15}$
Pump	Steel	$4.74\times 10^{4}$ ^a^	g	$2.77\times 10^{9}$ ^a^	$2.58\times 10^{9}$	$1.31\times 10^{14}$	$1.22\times 10^{14}$

^a^ Obtained from [57].

**Table 15:** The output of the emergoeconomic analysis of the case study.

Components	${\dot{U}}_{k}$ (sej/h)	${\dot{M}}_{D,k}$ (sej/h)	${\dot{M}}_{Total,k}$ (sej/h)	$m_{F,k}$ (sej/GJ)	$m_{P,k}$ (sej/GJ)	$r_{m,k}$ (%)	$f_{m,k}$ (%)
Evaporator	$1.35\times 10^{12}$	$5.49\times 10^{12}$	$6.84\times 10^{12}$	$2.77\times 10^{13}$	$3.39\times 10^{13}$	22.41	19.68
Turbine	$5.43\times 10^{12}$	$2.36\times 10^{12}$	$7.79\times 10^{12}$	$3.45\times 10^{13}$	$4.77\times 10^{13}$	38.43	69.71
Condenser	$1.77\times 10^{12}$	$4.37\times 10^{12}$	$6.14\times 10^{12}$	$3.45\times 10^{13}$	$5.33\times 10^{13}$	54.56	28.75
Pump	$3.16\times 10^{11}$	$1.69\times 10^{11}$	$4.85\times 10^{11}$	$4.77\times 10^{13}$	$1.04\times 10^{14}$	117.6	65.10
